# Recent Advances in the Analysis of Cold Tolerance in Maize

**DOI:** 10.3389/fpls.2022.866034

**Published:** 2022-04-12

**Authors:** Xuemei Zhou, Imran Muhammad, Hai Lan, Chao Xia

**Affiliations:** ^1^Maize Research Institute, Sichuan Agricultural University, Chengdu, China; ^2^Department of Chemistry, Punjab College of Science, Faisalabad, Pakistan; ^3^State Key Laboratory of Crop Gene Resource Exploration and Utilization in Southwest China, Sichuan Agricultural University, Chengdu, China

**Keywords:** maize, cold tolerance, QTL, GWAS, transcriptome, long-distance signaling

## Abstract

Maize (*Zea mays* L.) is an annual grass that originated in tropical and subtropical regions of the New World. Maize is highly sensitive to cold stress during seed gemination and the seedling phase, which can lead to reductions in plant vigor and grain production. There are large differences in the morphological and physiological changes caused by cold stress among maize varieties. In general, cold tolerant varieties have a stronger ability to maintain such changes in traits related to seed germination, root phenotypes, and shoot photosynthesis. These morphological and physiological characteristics have been widely used to evaluate the cold tolerance of maize varieties in genetic analyses. In recent years, considerable progress has been made in elucidating the mechanisms of maize in response to cold tolerance. Several QTL, GWAS, and transcriptomic analyses have been conducted on various maize genotypes and populations that show large variations in cold tolerance, resulting in the discovery of hundreds of candidate cold regulation genes. Nevertheless, only a few candidate genes have been functionally characterized. In the present review, we summarize recent progress in molecular, physiological, genetic, and genomic analyses of cold tolerance in maize. We address the advantages of joint analyses that combine multiple genetic and genomic approaches to improve the accuracy of identifying cold regulated genes that can be further used in molecular breeding. We also discuss the involvement of long-distance signaling in plant cold tolerance. These novel insights will provide a better mechanistic understanding of cold tolerance in maize.

## Introduction

Maize (*Zea mays* L.) is one of the world's most important cereal crops for food, economy, and feed (Wang et al., 2020a). Maize is particularly susceptible to cold injury as it is a crop that requires high temperatures due to its tropical/subtropical origins (yyGreaves, 1996). Although studies have shown that maize is susceptible to the effects of low temperature during grain filling (Chen and Tang, 2012), cold stress mainly affects seed germination, seedling development, and growth at the seedling phase, eventually leading to reduced grain production (Li et al., 2019b). Cold damage in early spring is one of the main meteorological disasters that can befall maize production, especially in northern regions and high altitude areas in mountainous regions (Zhang, 2014).

Cold reduces both the seed germination rate and seedling vigor (Zhang et al., 2020). When maize seeds are exposed to low temperatures during the water absorption phase (imbibition), the permeability of cell membranes is impaired, resulting in the loss of cellular components (Hussain et al., 2018). Cold may also damage the ultrastructure of the embryonic root meristem cells and impair root development (Farooq et al., 2009). Maize seedling growth slows down considerably when the temperature falls below 10°C, and it ceases at temperatures between 6 and 8°C (Peng et al., 2016). The cells and tissues of the seedling can be irreversibly damaged at lower temperatures. Low temperatures during the seedling phase can reduce plant height, root length, the ability of the roots to absorb mineral nutrients, leaf chlorophyll content, and the net photosynthetic rate in seedlings. This will eventually result in growth inhibition, leaf yellowing, wilting, and necrosis, or even death of the seedlings (Chen and Tang, 2012; Yang et al., 2018). Cold stress can not only reduce the emergence rate and seedling vitality of maize directly, but can also affect plant health indirectly by increasing the chances of infection by soil bacteria (Juurakko et al., 2021). These direct and indirect impacts can both acutely reduce the yield and quality of the maize crop. Improving cold tolerance in maize will allow early sowing to withstand cold snaps that occur in early spring. Early sowing can result in an extended vegetative period, thus allowing the plants to accumulate additional biomass (Aydinoglu, 2020).

Detailed cold tolerance mechanisms have been investigated in other plant species, such as *Arabidopsis* [*Arabidopsis thaliana* (L.) Heynh.] and rice (*Oryza sativa* L.; Farhangi-Abriz and Torabian, 2017; Ding et al., 2019; Ritonga and Chen, 2020). In recent years, considerable progress has been made in elucidating the mechanisms by which maize responds to cold stress. Although three very recent reviews have discussed the efficacy of genetic and genomic approaches used to assess maize cold tolerance (Frascaroli and Revilla, 2018; Sowiński et al., 2020; Gillani et al., 2021), a comprehensive review of this subject is still lacking. Here, we have summarized the recent progress in molecular, physiological, genetic, and genomic analyses of cold tolerance in maize in order to provide a theoretical basis for molecular breeding of cold tolerance in maize.

## The Cold-Responsive Molecular Network in *Arabidopsis*

**Figure 1:**
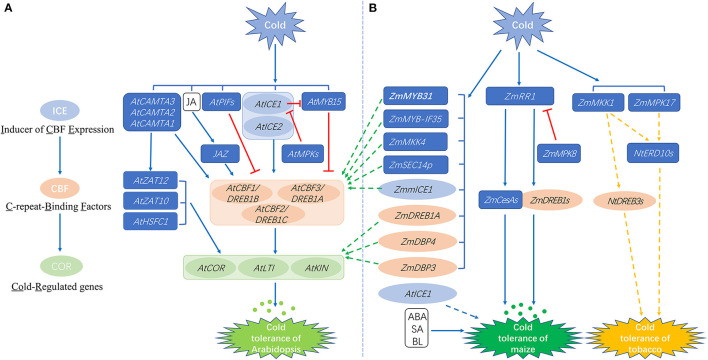
Schematic diagram of the cold-responsive molecular networks in Arabidopsis **(A)** and maize **(B)**. The eight green dashed arrows that point to **(A)** from **(B)** indicate that the maize genes were functionally verified in transgenic *Arabidopsis* plants. The orange dashed arrows in **(B)** indicate that the maize genes were functionally studied in tobacco. The blue dashed arrow in **(B)** indicates that the Arabidopsis gene *AtICE1* was transformed into forage maize. The colored ellipses represent molecular elements that belong to the ICE-CBF-COR pathway. Small dots represent osmotic substances. Brackets encompass genes with the same induction level. Straight and dashed arrows represent positive regulation, whereas lines ending with a bar represent negative regulation. At, *Arabidopsis thaliana* L.; Zm, *Zea mays* L.; Zmm, *Zea mays* ssp. mexicana L.; Nt: *Nicotiana tabacum* L.; CAMTA, Calmodulin-binding transcription activator; ZAT, Zinc-finger transcription factor; HSFC, Heat shock transcription factor C; JAZ, Jasmonate ZIM-domain; DREB, Dehydration responsive element binding factor; LTI, Low temperature induced; KIN, Cold inducible; MPK, Mitogen-activated protein kinase; SEC14P, Sec14-like protein; RR, Response regulator; DBP, Dehydration responsive element binding protein; CesA, Cellulose synthase; MKK, Mitogen-activated protein kinase kinase; ERD, Early response to dehydration; JA, Jasmonic acid; ABA, abscisic acid; SA, Salicylic acid; BL, Brassinolide.

The cold-responsive pathways of *Arabidopsis* have been the subject of intensive studies. Briefly, when *Arabidopsis* plants are exposed to cold stress, some cold-responsive inorganic substances activate transcription factors (TFs) through signal transduction pathways. Activated TFs bind to cis-elements present in downstream cold-responsive genes to activate their expression and induce cold tolerance in the plants (Li et al., 2019a). The signal pathway with CBF (C-repeat-binding factor) transcription factors as the core elements is mainly involved in regulation of the cold response (Rihan et al., 2017; shown in Figure 1A). There are three members of the CBF gene family in *Arabidopsis*: *CBF1/DREB1B* (*dehydration responsive element binding factor 1B*), *CBF2/DREB1C*, and *CBF3/DREB1A* (Liu et al., 2018). Overexpression of *AtCBF1, AtCBF2*, and *AtCBF3* in *Arabidopsis* and other plant species is reported to significantly improve plant cold tolerance (Liu et al., 2018, 2020; Ding et al., 2019). The *CBF* genes have been cloned from other plant species such as rice, *Brassica rapa*, wheat and maize, indicating that the *CBF* genes show an important association with the cold response in plants (Liu et al., 2018, 2020; Ding et al., 2019).

Overexpression of *CBF* genes significantly induces the expression of *COR* (*Cold-regulated*) genes in plants (light green ellipses in Figure 1A). *COR* refers to a class of genes regulated by cold stress, such as *LTI* (*LOW TEMPERATURE INDUCED*) and *KIN* (*COLD INDUCIBLE*). Some of these genes encode key enzymes involved in synthesizing osmotic substances, and are associated with the accumulation of cryoprotective proteins and soluble sugars to normalize cellular osmotic pressure and provide protection from freezing damage (Shi et al., 2018; Ding et al., 2019; small dots in Figure 1A). The CBF protein recognizes CRT/DRE (C-repeat/ Dehydration Responsive Element) motifs in the promoters of *COR* genes and induces their expression to enhance cold tolerance (Lu et al., 2017). The removal of the CBF-CRT/DRE regulatory module of AtCBF2 protein reduces the level of cold resistance in plants (Park et al., 2015).

The expression of *CBF* genes is regulated by both transcriptional activators and repressors (indicated by arrows and lines with a bar in Figure 1A). The positive regulatory genes include *AtICE1* (*inducer of CBF expression 1*), *AtICE2*, and *CAMTA* (*calmodulin-binding* transcription *activator*; Chinnusamy et al., 2007; Ding et al., 2015). The transcriptional inhibitors which are directly involved in inhibiting cold-induced CBF activation include AtMYB15 and the AtPIFs (phytochrome interacting factors; Shi et al., 2018; red lines with a bar in Figure 1A). The AtICE1 protein interacts with the MYC-binding sequences present in the promoters of *CBF* genes to increase their expression during cold stress (Chinnusamy et al., 2003). Over-expression of *AtICE2* has been shown to improve cold tolerance and the expression of *AtCBFs* in *Arabidopsis* (Fursova et al., 2009). The CAMTA3 protein binds to a CAMTA DNA regulatory motif, vCGCGb, located in a region of the promoter of *AtCBF* genes and functions together with CAMTA1 and CAMTA2 to promote the expression of *CBF* genes and cold tolerance in plants (Doherty et al., 2009; Kim et al., 2013). AtMYB15 and AtICE1 form a protein complex that binds to MYB recognition sites in the promoters of *CBF* genes. Overexpression of *AtMYB15* results in reduced expression of *CBF* genes, whereas loss-of-function of *AtMYB15* leads to increased expression of *CBF* genes (Agarwal et al., 2006). Furthermore, it has been recently discovered that redox changes mediated by cold stress can induce structural transformations and functional activation of CBF proteins (Lee et al., 2021).

At present, the ICE-CBF-COR cold responsive pathway is a well-accepted defense mechanism to cope with cold stress, but only some of the *COR* genes are regulated by CBF (Park et al., 2015). Some other transcription factors, including HSFC1 (Heat shock transcription factor C1), AtZAT10, and AtZAT12, are also capable of inducing *COR* gene expression under cold stress, and may co-regulate cold signal transduction with CBF (Park et al., 2015). Plant hormone JA (jasmonic acid) also positively regulates the CBF signal by mediating the interaction between the JAZ1/4 protein and ICE1/2, thereby regulating the transcriptional activity of ICE proteins and the expression of *CBF1-3* (Hu et al., 2013). Taken together, these findings indicate that the cold regulatory network is very complicated in its functioning and needs to be seen in a broader sense so that it can be better understood.

## The Cold-Responsive Molecular Network in Maize

The ICE-CBF-COR pathway has also been investigated in maize, in order to understand its involvement in cold tolerance in maize (as shown by ellipses in Figure 1B). However, the functional verification of most of these maize genes, as well as other cold-responsive genes, were determined by expression in heterologous species such as *Arabidopsis* or tobacco, and not in maize (green and orange arrows in Figure 1B). Four maize *CBF* TF genes, including*ZmDREB1A, ZmDREB2A, ZmDBP3*, and *ZmDBP4*, are induced by cold stress in maize. Overexpression of *ZmDREB1A, ZmDBP3*, and *ZmDBP4* in *Arabidopsis* induced the overexpression of cold-inducible genes, resulting in enhanced cold tolerance (Qin et al., 2004; Wang and Dong, 2009; Wang et al., 2011). The overexpression of *ZmDREB2A* was found to enhance thermotolerance, but not cold tolerance (Qin et al., 2007). A low temperature-associated gene, *ZmmICE1*, has been isolated from *Zea mays ssp. mexicana* L., a close relative of maize, and ectopic expression of *ZmmICE1* in the *Arabidopsis ice1-2* mutant was observed to be associated with enhancing plant cold tolerance (Lu et al., 2017). When transformed with the *Arabidopsis AtCBF1* gene, plants of the forage maize line SAUMZ1 showed reduced relative electrolyte leakage compared to wild type plants, and this resulted in improved cold tolerance (Xiang et al., 2012; blue dashed arrow in Figure 1B). These studies indicate that ICE-CBF-COR is a conserved pathway in various plant species. Other than the *ICE* and *CBF* genes reviewed above, there are many other regulators, such as transcription factors and protein kinases related to cold tolerance, that have been functionally characterized (Weckwerth et al., 2015; Erpen et al., 2018; Kimotho et al., 2019; Wang and Fu, 2019).

### *ZmMYB* Family Genes

The MYB family is one of the largest TF families in plants, and it was named for the conserved MYB domain that is present in all eukaryotic MYB TFs (Katiyar et al., 2012). MYB TFs play important roles in the tolerance to various stresses in plants, including cold stress (Wu et al., 2019). The *Arabidopsis CBF* gene promoters contain a MYB recognition sequence and can be activated by MYB TFs (Li et al., 2019a). Unlike *AtMYB15* in Arabidopsis, which is a negative regular of *CBF* gene transcription, two characterized maize MYBs are positive regulators of *CBF* genes (Figure 1B). Expression of the maize *ZmMYB31* gene, which encodes an R2R3-MYB transcription factor, is induced at low temperature. Overexpression of *ZmMYB31* in *Arabidopsis* was found to upregulate the expression of *Arabidopsis CBF* genes, thereby enhancing the resistance of transgenic *Arabidopsis* plants to low temperature and oxidative stress (Li et al., 2019a). Meng and Sui (2019) isolated another nucleus-located R2R3-MYB transcription factor, ZmMYB-IF35. Low temperature induced the expression of *ZmMYB-IF35* in the cold-tolerant maize inbred line M54. Transgenic *Arabidopsis* plants overexpressing *ZmMYB-IF35* showed improved cold tolerance, higher antioxidant enzyme activity, reduced levels of reactive oxygen species (ROS), and less ion leakage. *ZmMYB-IF35* expression also positively regulated the expression of stress-related genes such as *AtCBF2, AtCBF3, AtCOR1*, and *AtCOR2*. Similar to the functions of maize *ZmMYB* genes, overexpression of a rice R2R3-MYB gene, *OsMYB2*, was found to improve cold tolerance, indicating that OsMYB2 is also a positive regulator of cold tolerance in rice (Yang et al., 2012). Further analysis of the expression patterns of 46 *ZmMYB* genes under different abiotic stress showed that 22 of these genes respond to different stress treatments. Among them, there were six *ZmMYB* genes that responded to cold treatment, but only *ZmMYB53* expression was exclusively induced by cold stress (Chen et al., 2018).

### Protein Kinase Family Genes

MAPK or MPK (mitogen-activated protein kinases) proteins, a family of serine/threonine protein kinases, are involved in many important processes including stress signal transduction and development (Kong et al., 2013). The MAPK cascade pathway is a ubiquitous signal transduction module in eukaryotes that transmits biological signals from receptors to target molecules through a variety of intracellular and extracellular routes (Moustafa, 2014). The MAPK cascade component involves a three-kinase module, namely MAPK, MAPKK (MAPK kinase), and MAPKKK (MAPKK kinase; Xiang et al., 2021). The MAP kinase cascade can phosphorylate AtICE1 to promote its degradation, and thus is involved in the regulation of cold tolerance in *Arabidopsis* (Zhao et al., 2017; Figure 1A). In maize leaves, *ZmMPK5* was found to be involved in the recovery of plants from cold stress (Berberich et al., 1999). Kong et al. (2011) isolated *ZmMKK4*, a group C MAPKK gene, from the root system of maize variety “Zhengdan 958,” and expression of the *ZmMKK4* transcript was found to be up-regulated by low temperature exposure. Overexpression of *ZmMKK4* in *Arabidopsis* increased the plants' cold tolerance, which showed that *ZmMKK4* is a positive regulator of cold tolerance in maize (indicated by the green arrow in Figure 1B). Pan et al. (2012) identified *ZmMPK17*, a group D MAPK gene, that was induced by cold stress. The overexpression of *ZmMPK17* enhanced cold tolerance in tobacco (*Nicotiana tabacum* L.) plants by affecting the antioxidant defense system. Cai et al. (2014) isolated and identified *ZmMKK1*, a group A MAPKK gene, from “Zhengdan 958”. Ectopic expression of *ZmMKK1* in tobacco enhanced its cold tolerance, suggesting that *ZmMKK1* is also involved in the response of plants to low temperature.

A very recent study identified a new cold regulation pathway, ZmMPK8-ZmRR1-ZmDREB1.10/ZmCesA2, in maize (Zeng et al., 2021; Figure 1B). The *ZmRR1* (*type-A Response Regulator 1*) transcript level is slightly decreased, whereas the ZmRR1 protein level is increased by cold treatment of maize seedlings at 4°C. Overexpression of *ZmRR1* leads to enhanced cold tolerance by accumulating and inducing the expression of the downstream genes, *ZmDREB1.10* and *ZmCesA2* (*Cellulose synthase 2*), suggesting that *ZmRR1* acts as a positive regulator of maize cold tolerance. The ZmMPK8 protein, a negative regulator of cold tolerance, phosphorylates ZmRR1 at Ser15. A natural variation of *ZmRR1* with a 45-bp deletion that encompasses Ser15 prevents its phosphorylation by ZmMPK8. At present, the ZmMPK8-ZmRR1-ZmDREB1.10/ZmCesA2 pathway is the best characterized cold regulation pathway in maize, and it provides an in-depth understanding of the molecular mechanism underlying cold tolerance in maize.

To date, functional investigations of many cold responsive genes have been performed in maize, but the functions of many of these genes were studied by overexpression in model plant species. As shown by the green and orange dashed arrows in Figure 1B, a total of eight maize genes were transformed into *Arabidopsis*, and two maize genes were transformed into tobacco. Only one study investigated the molecular function of *ZmRR1, ZmMPK8*, and their downstream genes *ZmDREB1.10* and *ZmCesA2*, by either mutating or over-expressing the corresponding genes in maize. Transformation of maize genes into maize plants not only provides stronger evidence for gene functions, but also enables the straightforward elucidation of molecular pathways to provide in-depth biological insights. Furthermore, such native transformation would provide potential genetic resources for improving cold tolerance in maize.

## Physiological Acclimation and Seed Treatments to Relieve Damage in Maize Due to Cold Stress

Cold stress can induce a series of physiological responses in maize, such as the adjustment of osmotic substances, accumulation of ROS, disruption of hormonal homeostasis, impaired uptake of mineral nutrients, and a decrease in photosynthesis. To survive under such unfavorable conditions, plants need to maintain cellular function and integrity by stabilizing the cell membranes and biologically active proteins in order to sustain basic physiological activities (Ritonga and Chen, 2020). Even though plants have developed mechanisms by which they can to acclimate to cold temperatures, several strategies have been developed to reduce the effects of cold stress on maize seedlings.

### Adjustment of Osmotic Substances

Osmotic adjustment is one of the most important physiological mechanisms employed by plants to cope with many types of stresses. Under adverse conditions, osmotic adjustment substances help to maintain cell turgor and the capacity of tissues to retain water, and the contents of osmotic adjustment substances are positively correlated with plant stress resistance (Farhangi-Abriz and Torabian, 2017). The accumulation of osmotic adjustment substances can reduce the water potential in plant cells, prevent water outflow, and at the same time protect the structure of macromolecules in the cell (Li et al., 2013). The main osmotic adjustment substances in plants include soluble sugars, soluble proteins, and free proline (Zhang, 2014).

Soluble sugars are strongly hydrophilic, and they can reduce the stress damage to plant cells by reducing the water potential and maintaining the activity of some biological macromolecules (Guan et al., 2009). Soluble proteins can scavenge ROS (reactive oxygen species) and stabilize cell membrane structure (Nadarajah, 2020). Proline is a hydrophilic amino acid, and free proline functions to stabilize the metabolic process in the cytoplasm (Guan et al., 2009). Ma et al. (2015a) found that cold stress induces the expression of *ZmP5CS1*, a key gene for proline synthesis in immature maize embryos. Some studies have reported that low temperature treatments increase the contents of soluble sugars, proline, and soluble proteins in the seedlings of many maize varieties. The relative increases were larger in the varieties with strong cold tolerance compared to the varieties with relatively weaker cold tolerance. The lower the temperature, the more significant the difference between varieties (Li and Fang, 2018; Zhao et al., 2020). In conclusion, cold tolerant maize can maintain the water absorption capacity of cells and can reduce cold damage by accumulating higher levels of osmotic adjustment substances in response to cold stress.

### Antioxidant Enzyme System and MDA Production

Cold stress can destroy cellular homeostasis and cause the accumulation of high levels of ROS in plant cells. Excessive levels of ROS can be harmful to the cell membrane system because of lipid peroxidation (Nadarajah, 2020). Plant cells can induce the expression of a protective system to resist the threat posed by ROS. The protective enzymes within a plant cell, such as SOD (superoxide dismutase), CAT (catalase), POD (peroxidase), and APX (ascorbate peroxidase), cooperate with one another to scavenge the reactive oxygen free radicals so as to maintain normal physiological metabolic activities as much as possible to avoid damage to cell components (Nadarajah, 2020). Maize cells can stabilize the membrane structures and reduce cell damage by constantly oxidizing the reactive oxygen free radicals. Many studies have shown that cold stress enhances the activities of SOD, POD, and CAT in maize. The activities of the three main antioxidant enzymes and the relative expression of related genes are positively correlated with the cold tolerance of inbred lines (Wei et al., 2014; Yang et al., 2016; Li and Fang, 2018). For example, an early study showed that low temperature induces the expression of the *APX* gene, and the APX activity in cold tolerant maize was found to be higher than in cold sensitive maize (Pinhero et al., 1997). One study showed that the activities of protective enzymes decreased in the three cold-tolerant and three cold-sensitive maize inbred lines under cold stress, but the relative decrease in the cold-tolerant inbred lines was less than that in the cold-sensitive inbred lines (Peng et al., 2016). These differences could be due to many reasons, but we can conclude that the antioxidant enzyme system in cold-resistant maize lines is generally stronger than that of cold-sensitive lines.

MDA (malondialdehyde) is the main product of peroxidation of polyunsaturated cell membrane lipids and is a marker for oxidative stress. MDA is a highly reactive compound that can restrain the activity of cell protective enzymes and aggravate membrane peroxidation. When the cell membrane is damaged, a large number of electrolytes flow out of the cell, resulting in a surge of electrolytes (Wang et al., 2006). Therefore, relative conductivity and MDA levels are negatively correlated with cold tolerance in maize lines (Zhang, 2014). Several studies have reported that with increasing time of exposure to cold, the relative conductivity and MDA content increases. The cell membranes of cold tolerant varieties were less damaged, and the relative conductivity and MDA contents increased to a lesser extent in maize lines that show strong cold tolerance (Zhang, 2014; Peng et al., 2016).

### Plant Hormones

Plants tightly regulate the levels of some hormones to cope with the changing environment (Lamers et al., 2020). ABA (abscisic acid) is an abiotic stress hormone that can improve the stress resistance of plants, and it participates in the regulatory response for a variety of abiotic stresses, including cold (Qin et al., 2021). The ABA contents in the roots of cold-tolerant lines were significantly higher than in the roots of cold-sensitive lines. Exogenous ABA treatment can promote the ability of maize seeds to germinate at low temperature (Zhang, 2011). GA (gibberellic acid) is also considered to play a role in plant cold resistance, but its effect is not as obvious as that of ABA (Eremina et al., 2016; Rihan et al., 2017). Studies have shown that the GA and IAA (indole-3-acetic acid; auxin) contents in the roots of maize seedlings decrease under cold stress, while the ABA content gradually increased (Janowiak et al., 2002; Zhang, 2011; Wei et al., 2014).

SA (salicylic acid) is another important plant hormone that is involved in cold tolerance in several plant species including maize, and its direct physiological effect is the change in the antioxidant enzyme activity of plants (Farooq et al., 2009). At low temperature, the increase in endogenous SA biosynthesis is closely related to the increase in antioxidant enzyme activity in maize seeds and during seedling growth (Wang et al., 2013). Cold stress in maize seedlings was not moderated by treating the seeds with SA (Gómez-Muñoz et al., 2018), but SA+H_2_O_2_ treatment at low temperature shortened seed germination time, improved seedling vigor, reduced cold damage to maize seeds, increased the activities of antioxidant enzymes and the expression levels of the corresponding genes, and thus improved cold tolerance (Li et al., 2017). Exogenous application of BL (brassinolide) also increased the germination rate, reduced cold damage to maize seedlings and caused an increase antioxidant enzyme activity, all of which resulted in an increase in plant biomass (Sun et al., 2020). Thus, manipulation of plant hormones appears to be an efficient way to alleviate the damage caused by cold temperature and to ensure the growth of seedlings at later stages of development.

### Mineral Nutrients

Cold tolerance in plants is highly correlated with mineral nutrient levels (Waraich et al., 2012). The inhibition of growth in maize from cold stress is at least partly caused by indirect damage due to the decreased uptake of nutrients from the soil (Gómez-Muñoz et al., 2018). For example, the absorbance of K and Pi (orthophosphate) by maize roots is particularly affected by cold soil temperature (Bravo et al., 1981). Seed treatment with Mn/Zn can mitigate the negative effects of cold stress, and it resulted in increased biomass production in high-P soil but not in low-P soil, indicating that the absorbance of P is relieved by Mn/Zn treatment (Gómez-Muñoz et al., 2018). In addition, both Mn and Zn are key co-factors of several enzymes that are involved in the detoxification ROS caused by cold stress. Application of Si (silicon) is also a useful strategy to improve cold tolerance in maize seedlings during the early growth stage. The beneficial effects of Si seed treatments included the restoration of hormonal balances that were disrupted by cold stress and maintaining homeostasis of other micronutrients (Moradtalab et al., 2018). Therefore, appropriate plant nutrition is a useful strategy to alleviate the cold stress.

### Photosynthesis

Cold stress significantly decreases photosynthesis in maize (Fracheboud et al., 2002). This decrease in photosynthesis may result from many factors, such as impaired chloroplast development, changes to the pigment composition, damage of the PSII reaction centers, and reduced activity of carbon cycle enzymes. The reduced photosynthetic ability at low temperatures also reflects the cold resistance of maize (Hund et al., 2008). Fracheboud et al. (2002) found that the chlorophyll a/b ratio in the cold tolerant lines was higher than in cold sensitive lines. Duran Garzon et al. (2020) showed that cold tolerance is related to higher chlorophyll content, higher G6PD (glucose-6-phosphate dehydrogenase) activity, and a higher sucrose-to-starch ratio. Many studies have used photosynthesis-related traits to quantify cold tolerance in maize. These studies are discussed below and are given in Tables 1, 2.

**Table 1:** Quantitative trait loci (QTLs) for traits related to cold tolerance in maize.

**References**	**Population**	**Number of families or inbred lines**	**Traits or indicators**	**Number of QTLs**	**Chr**.
Fracheboud et al. (2002)	RILs (Ac7643 × Ac7729/TZ)	233	Fv/Fm, ΦPSII, SR, etc.	18	1, 2, 3, 4, 6, 7, 9
Fracheboud et al. (2004)	F2:3 (ETH-DH7 × ETH-DL3)	266	CFP, SDW, SPAD, etc.	19	1, 2, 3, 4, 6, 8
Hund et al. (2004)	F2:4 (Lo964 × Lo1016)	168	GI, Fv/Fm, RL, etc.	60	1, 2, 3, 4, 5, 6, 7, 8, 9, 10
Jompuk et al. (2005)	F2:3 (ETH-DH7 × ETH-DL3)	226	CFP, SPAD, LA, etc.	29	1, 2, 3, 4, 5, 6, 7, 8, 9, 10
Presterl et al. (2007)	DH (SL × TH)	720	LC‘, LP, and FD	18	1, 2, 3, 4, 5, 6, 7, 8, 9, 10
Rodríguez et al. (2008)	RILs (B73 × Mo17)	302	LC	2	3, 6
Guerra-Peraza et al. (2012)	RILs (B73 × Mo17)	295	Fq'/Fm', Fv/Fm, SPAD, etc.	19	4, 5, 6, 7, 8, 9
Rodríguez et al. (2014)	F2:3 (EP42 × A661)	210	DW, ΦPSII, TAC, etc.	4	2, 4, 8
Shi et al. (2016)	RILs (Yu82 × Shen137 and Yu537A × Shen137)	420	Gp, GI, MGT, etc.	26	1, 2, 3, 4, 5, 6, 7, 8, 9
Hu et al. (2016)	RILs (B73 × Mo17)	243	LTGR and LTPRL	12	4, 5, 6, 7, 9
Yan et al. (2017)	F2:3 (K932 × Mei C)	207	LRD, WCS, RRS, etc.	7	1, 2, 3
Li et al. (2018)	F2:3 (220 × PH4CV, 220 × Y1518 and P9-10 × PH4CV)	650	ER, GI, RL, etc.	43	1, 2, 3, 4, 5, 8, 9, 10
Yi et al. (2020)	RILs (MAGIC)	406	RLCC, Fv/Fm, SDW, etc.	62	1, 2, 3, 4, 5, 6, 7, 8, 9, 10
Goering et al. (2021)	RILs (B73 × Mo17)	97	CC, LC, and TD	2	1, 5
Jin et al. (2021)	Two genetic populations	290	PA	12	2, 3

*MAGIC, Multi-parent advanced generation intercross population; DH, Doubled haploid; Fv/Fm, Maximum quantum efficiency of photosystem II; ΦPSII, Quantum yield of photosystem II; SR, stomatal resistance; CFP, Chlorophyll fluorescence parameters; SDW, Shoot dry weight; SPAD, leaf greenness; GI, Germination index; RL, Root length; LA, Leaf area; LC', Leaf chlorosis; LP, Leaf purpling; FD, Frost damage; LC, Leaf color; Fq'/Fm', Operating quantum efficiency of photosystem II; DW, Dry weight; TAC, Total anthocyanin content; Gp, Germination percentage; MGT, Mean germination time; LTGR, Low-temperature germination rate; LTPRL, Low-temperature primary root length; LRD, Leaf rolling degree; WCS, Water content in shoots and leaves; RRS, Ratio of root-to-shoot; ER, Emergence rate; RLCC, relative leaf chlorophyll content; CC, chlorophyll concentration; TD, Tissue damage; PA, Peroxidase activity; Chr, chromosome number*.

**Table 2:** SNPs linked to traits related to the bud and seedling stages in maize.

**References**	**Population**	**Number of families**	**Traits or indicators**	**Number of**	**Number of SNPs**	**Chr**.
		**or inbred lines**	**genotyped SNPs**
Strigens et al. (2013)	Diversity panel	375	Fv/Fm, SPAD, LA, etc.	56,110	19	1, 4, 5, 6, 7, 10
Huang et al. (2013)	Association panel	125	RLN, RSL, RFSW, etc.	56,110	43	1, 2, 3, 4, 5, 6, 7
Yan et al. (2017)	TAMP(S-Mo17)	338	LRD, WCS, RRS, etc.	556,809	19	1, 2, 3, 4, 6, 10
Hu et al. (2017)	Association panel	282	RDT50, RGI, RGR, etc.	2,271,584	17	1, 2, 4, 6, 7, 9
Zhang et al. (2020)	Association panel	222	RGR, RGL, RRL, etc.	40,697	30	1, 2, 3, 4, 5, 6, 7, 8, 9, 10
Yi et al. (2021)	Association panel	836	DE, EV, RLCC, etc.	156,164	32	1, 2, 3, 4, 5, 6, 7, 8, 9, 10
Zhang et al. (2021)c	Association panel	300	FG, RL, RRS, etc.	43,943	15	1, 2, 3, 4, 5, 7, 8, 10
Jin et al. (2021)	Two genetic populations	290	PA	24,860,241 + 5,759,868	4	3

*TAMP, Testcrossing association mapping population; Fv/Fm, Maximum quantum efficiency of photosystem II; SPAD, leaf greenness; LA, Leaf area; RLN, Relative leaf number; RSL, Relative shoot length; RFSW, Relative fresh shoot weight; LRD, Leaf rolling degree; WCS, Water content in shoots and leaves; RRS, Ratio of root-to-shoot; RDT50, Relative days to 50% Germination; RGI, Relative germination index; RGR, Relative germination rate; RGL, Relative germ length; RRL, Relative radicle length; DE, Days to emergence; EV, Early vigor; RLCC, relative leaf chlorophyll content; FG, Germination rate at 5 d; RL, Root length at 10 d; PA, Peroxidase activity; Chr, chromosome number*.

In general, cold tolerant maize may have stronger ability to accumulate more osmotic adjusting substances, stronger antioxidant enzyme system to stabilized ROS, stronger ability to maintain hormonal homeostasis, higher absorbance of mineral nutrient, higher photosynthetic ability, etc.

## Genetic and Genomic Approaches to Dissect Cold Tolerance in Maize

Cold tolerance in maize is a quantitative agronomic trait that is controlled by multiple genes (Turk et al., 2019). In the past few years, a number of genetic and genomic approaches have been applied to dissecting the components of this complex trait.

### Quantitative Genetics

Quantitative genetic analyses of cold tolerance in maize have so far shown that heterosis, general and specific combining abilities, reciprocal maternal and non-maternal effects, additive effects, genotype, growth stage, and environmental factors are all involved in the expression of cold tolerance in maize. For example, Hodges et al. conducted two studies to explore the cold tolerance of maize at both germination and seedling stages using twelve hybrids derived from a complete diallel of four inbred lines (Hodges et al., 1997a,b). The results showed that the seed germination rate, the activities of CAT and APX in leaves at the 3-leaf stage, and the general and the special combining ability of the dry matter weight of leaves at the 4-leaf stage were significantly different. Revilla et al. (2000) proposed that cold tolerance has additive, dominant, and maternal effects. The experiment of Yan et al. (2017) showed that there was significant heterosis in maize cold tolerance at the seedling stage. Neta et al. (2020) used three cold-tolerant lines and three cold-sensitive lines to carry out partial diallel cross experiments to analyze the heterosis, general and specific combining abilities, reciprocal maternal and non-maternal effects, as well as the expression of CAT, APX, SOD and other genes. Results showed that there was heterosis and a reciprocal effect for germination under cold stress, and that the non-additive genes were more important. The genes that control cold tolerance depend on the particular materials used and the traits studied (Frascaroli and Revilla, 2018). Each growth stage of maize, including germination, emergence, and early seedling growth, is controlled by an independent genetic model; therefore, cold tolerance might also be regulated independently in the different growth stages (Frascaroli and Revilla, 2018). To make the situation more complicated, some studies have also reported that there are interactions between genes and the environment in the expression of cold tolerance in maize (Presterl et al., 2007).

The studies cited above show that the genetics of cold tolerance in maize is very complicated. However, with the broad application of genomic tools, considerable progress has been made in recent years in the identification of genetic loci and genes that are associated with maize cold tolerance. The information from these studies will facilitate a deeper understanding of the genetic mechanisms that control maize cold tolerance at the molecular level.

### QTL Mapping

QTL (quantitative trait locus) mapping is a powerful tool for the identification and manipulation of loci underlying important and complex traits in agricultural crops. Recent studies have used different mapping populations, such as F_2:3_ families, RILs (recombinant inbred lines), and BC (back cross) populations, to study the QTLs that control cold tolerance in maize (Table 1). Most of these segregating populations were constructed by crossing cold-sensitive and cold-tolerant inbred lines in order to increase the relative degree of variation in the cold-response phenotypes. Several cold responsive physiological traits, such as seed germination rate, root phenotypes, and seedling photosynthesis, were measured under both normal and cold conditions (Table 1). These studies identified many reliable molecular marker loci associated with QTLs or genes that regulate cold tolerance and could be further used for breeding of cold tolerant inbred lines or hybrids.

Fracheboud et al. (2002) performed a QTL analysis of five traits related to the function of the photosynthetic apparatus at low temperature using a set of 233 maize RILs. They identified 18 QTLs that were significantly correlated with the target traits, of which the main QTL for leaf development at low temperature was the main QTL for pigment composition. Fracheboud et al. (2004) used an F_2:3_ population derived from the cross ETH-DH7×ETH-DL3 and detected 19 QTLs controlling chlorophyll fluorescence at low temperature. The major QTL for photosynthetic cold tolerance in maize seedlings is located on chromosome 6. Using the same population, Jompuk et al. (2005) detected a major QTL for cold tolerance located on chromosome 6 that corresponded to chlorophyll fluorescence and chlorophyll content. Hund et al. (2004) used an F_2:3_ population constructed from the cross Lo964×Lo1016 to map QTLs for root and shoot development in maize seedlings under cold conditions, and found a dominant QTL located on chromosome 5. Presterl et al. (2007) performed QTL mapping on 720 DH (doubled haploid) lines and found a total of 18 QTLs associated with leaf chlorosis, leaf purpling (anthocyanin), and frost damage at low temperature. Rodríguez et al. (2008) performed QTL analysis by using an IBM (intermated B73×Mo17) population. None of the QTLs was identified under normal growth conditions, but two QTLs significantly correlated with leaf color at low temperature were located on chromosomes 3 and 6, and these two OTLs explained 14.2% of the phenotypic variation and 28.2% of the genetic variation. Guerra-Peraza et al. (2012) used the IBM302 population for QTL analysis and discovered a major QTL for photosynthesis-related traits on chromosome 5. The favorable allele of this QTL was contributed by Mo17 and appeared to be the major factor that explained the differential response of B73 and Mo17 to changes in the temperature at night. Rodríguez et al. (2013) used an F_2:3_ population derived from the cross of a cold susceptible (A661) with a cold tolerant (EP42) inbred line to detected genomic regions related to the cold-induced albino phenotype. A major QTL on chromosome 2 was identified that explained 14% of the phenotypic variation. Using the same F_2:3_ population, 10 QTLs related to photosystem traits were identified, with six of them at normal temperatures and four under cold conditions. A parallel meta-QTL analysis identified three genomic regions that regulate the development of maize seedlings at low temperatures (Rodríguez et al., 2014). Hu et al. (2016) performed a QTL analysis using 243 IBM Syn4 RILs and detected six QTLs associated with the low-temperature germination rate and six QTLs correlated with low-temperature primary root length. Of these, four pairs of QTLs were located in the same genomic regions. Yan et al. (2017) performed a QTL analysis with seedlings from an F_2:3_ population (207 lines) obtained by crossing a cold sensitive line (K932) with a cold tolerant (Mei C) inbred line. Their analysis resulted in the detection of seven QTLs controlling four cold-related traits, and one of the QTLs explained 10.55–25.29% of the phenotypic variation.

While the above-cited QTL analyses used “regular” populations, several studies have used “advanced” populations to improve the accuracy of the study. For example, Shi et al. (2016) constructed two connected RIL populations that shared one parental line and were able to identify 26 QTLs associated with seed vigor traits under low temperature conditions. Fourteen of these QTLs were further integrated into five mQTL regions through a meta-analysis. Two of the mQTL regions located on chromosomes 2 and 9 had *R*^2^ values >10% and were previously identified as QTLs for seed vigor traits. Using two cold-tolerant and two cold-susceptible inbred lines, Li et al. (2018) generated three connected F_2:3_ populations to detect QTLs related to seed germination ability at low temperature. A total of 43 QTLs and three mQTL regions were detected. Yi et al. (2020) used 406 lines from a multi-parent advanced generation intercross (MAGIC) population and found 858 SNPs grouped in 148 QTLs that were significantly associated with cold tolerance-related traits, and most of the QTLs were located in specific regions, particularly bin 10.04.

Several studies combined QTL analyses with other genetic or genomic approaches such as GWAS (genome-wide association study) or transcriptome analysis to efficiently uncover further biological insights. From the same population used by Hu et al. (2016); Goering et al. (2021) selected a panel of 97 lines for QTL analysis of traits including chlorophyll concentration, leaf color, and tissue damage at low temperature. Two cold-related QTLs with high additive impact were detected. These authors further verified the two QTLs using transcriptome data and identified 13 candidate genes likely to be involved in controlling the cold responses. Recently, Jin et al. (2021) studied cold responses in maize using a joint analyses that combined QTL and GWAS. They first performed QTL mapping for POD activity using an F_2:3_ population (210 lines) derived from cold-tolerant (W10) and cold-sensitive (W72) lines, and detected 12 QTLs significantly associated with POD activity and cold tolerance. They then conducted GWAS on a natural population consisting of 80 backbone inbred lines and found that four SNPs were significantly associated with POD activity at low temperature (Table 2). Using a joint analysis of the QTL and GWAS results, *Zm00001d002729* was determined to be a potential cold tolerance gene. Overexpression of *Zm00001d002729* increased the cold tolerance of maize seedlings by increasing POD activity and decreasing the MDA content and relative conductivity (ion leakage).

Table 1 shows that the genomic loci involved in the regulation of cold tolerance are distributed on almost all of the maize chromosomes, but most of these QTLs have not been further fine mapped or functionally characterized as cold tolerant genes, which prevents more detailed studies of cold tolerant mechanism in maize. One major reason for this may be the huge amount of work involved in gene mapping in large populations. In recent years, joint analysis using advanced populations or genomic tools has become increasingly popular to narrow down the search for candidate genes. Such an approach was recently used successfully to identify the major-effect cold regulating gene *Zm00001d002729* (Jin et al., 2021), suggesting that it can be more widely applied in other studies.

### GWAS

Compared to biparental segregating populations, natural populations consisting of several hundred maize inbred lines have also been used for the genetic analyses of cold tolerance in GWAS (Frascaroli and Revilla, 2018). Although most of the cold-responsive physiological traits used in GWAS were similar to those in QTL analyses, including leaf color, root length, and seed germination-related traits (Tables 1, 2), GWAS offers increased mapping resolution and accuracy due to the higher level of genetic diversity in the mapping populations (Lipka et al., 2015).

Strigens et al. (2013) conducted the first GWAS in maize to dissect traits related to cold tolerance. Using a maize germplasm collection of 375 inbred genotypes with 56K SNPs (single nucleotide polymorphisms) GWAS identified 19 significant association signals that explained between 5.7 and 52.5% of the phenotypic variance for cold-related traits such as early growth and chlorophyll fluorescence. Huang et al. (2013) used 125 maize inbred lines to perform GWAS on 10 traits related to cold tolerance at the germination and seedling stages. A total of 43 SNPs were detected that were associated with cold tolerance and 40 candidate genes were predicted based on 31 of these SNPs. Yan et al. (2017) crossed the cytoplasmic male sterile parental line S-Mo17 with 338 different inbred lines to generate a test cross association mapping population. GWAS was performed on these 338 test crosses, and 19 significant SNPs associated with cold tolerance-related traits were detected. Hu et al. (2017) conducted GWAS on seed germination traits using 282 inbred lines of maize under normal and low temperature conditions. A total of 17 associated SNPs related to cold tolerance were identified, and seven of the SNPs were located in candidate genes. In a population of 222 maize inbred lines, Zhang et al. (2020) used GWAS to identify 30 SNPs related to cold tolerance during maize seed germination. Fourteen candidate genes directly related to the SNPs were found and further verified by gene expression analysis. Zhang et al. (2021) studied germination-related traits in 300 inbred lines under low temperature conditions. GWAS analysis revealed a total of 15 significant SNPs, and three genomic loci were repeatedly associated with multiple traits. Yi et al. (2021) evaluated a large panel of 836 maize inbreds, and GWAS analysis uncovered a total of 187 significant SNPs that could be integrated into 159 genomic regions that controlled seed emergence and traits related to early growth.

Despite its higher genetic mapping resolution, GWAS has not been without controversy. In particular, many of the cold-associated SNP markers identified in these studies were found to be located in the non-coding regions and generally thought to function in the regulation of gene expression. But which gene(s) do they regulate? There are large structural variations present in the genomes of different maize inbred lines, and the genetic loci predicted in the B73 genome may not represent all the genetic loci in other inbred lines. Hence, there may be some other unpredicted genes near or even located on cold-associated SNP markers, leading to inaccurate results.

### Transcriptomic Analyses

Transcriptome analyses are also widely used to understand the molecular responses of maize to cold stress and to mine for cold tolerant genes. Li et al. (2016) used RNA-seq analyses to compare the transcriptomes of a freezing tolerant (KR701) and a freezing sensitive line (Hei8834) before and after cold treatment at the seedling stage and identified 948 DEGs (differentially expressed genes). GO (Gene Ontology) analysis revealed that the terms “binding functions,” “protein kinase,” and “peptidase activity” were over-represented in the DEGs. Li et al. (2019b) used RNA-seq to analyze the gene expression of cold tolerant (M54) and cold sensitive (753F) inbred lines under cold stress at the seedling stage. More DEGs were found in M54 than in 753F after both 4 and 24 h of cold treatment, indicating that the cold-responsive signaling networks were more active in the cold tolerant line. Li et al. (2020) analyzed the transcriptome of maize B73 seedlings under different low temperature conditions. In this study, 5,358, 5,485, and 5,312 DEGs were detected in response to cold stresses of 4, 10, and 16°C, respectively, and the expression of five genes including Zm*DERB1* was significantly up regulated. Frey et al. (2020) selected 21 DH lines from a DH population (flint landrace “Petkuser Ferdinand rot”) based on their cold tolerance; 11 lines were cold resistant and 10 were cold sensitive. The transcriptomes of the 21 DH lines were analyzed after control and cold treatments. Here, 148, 3,254, and 563 DEGs were found to be related to cold treatment, cold tolerance, and growth rate at low temperature, respectively. Zhang et al. (2020) used RNA-seq to verify the correlation between the candidate genes and low-temperature tolerance and found 10 DEGs that were located in the linkage disequilibrium region of a GWAS analysis. Of these genes, two of them appear to regulate cold signal transduction and cell membrane fluidity. Li et al. (2021) analyzed transcriptomic changes in seeds of three sweet corn NILs and their parents under cold stress. A total of 20 DEGs were found to be highly related to low-temperature germination, and a gene encoding UDP-glucosyltransferase was hypothesized to be essential to cold germination in sweet corn. Waititu et al. (2021) conducted a comparative analysis of the transcriptomes of seedlings of 24 cold-tolerant and 22 cold-sensitive inbred lines under cold stress. A total of 2,237 DEGs were identified, which included 147 TFs belonging to 32 families such as MYB, ERF, NAC, WRKY, bHLH, MIKC MADS, and C2H2. Yu et al. (2021) studied the leaf transcriptomic response of two maize inbred lines with contrasting cold tolerance levels under a time series cold treatment. The results showed that cold tolerance in line B144 is due to active mediation of stomatal opening and protection of photosystem II from photooxidation by upregulating the expression of genes for D1 proteins, while the sensitive line Q319 was unable to close its stomata in response to cold.

In addition to RNA-seq analyses, other genomic tools have also been used to study the transcriptomic changes in maize to cold. Rodríguez et al. (2013) used microarray hybridization on the chlorophyll-less and chlorophyll-containing sections of leaves of maize inbred line A661 which shows a cold-induced albino phenotype. A total of 1,002 differentially expressed transcripts were identified between the two sections, and these DEGs were classified into 23 categories including genes in the tetrapyrrole biosynthesis pathway and photosynthesis. Di Fenza et al. (2017) conducted microarray analysis with four varieties, with two cold-tolerant and two less cold-tolerant lines, to identify genes that were differentially expressed under chilling conditions. A total number of 64 DEGs were identified in the two chilling-tolerant varieties, while no significant changes in expression were observed in less cold-tolerant lines. Another study used cDNA-AFLP to analyze the gene expression changes in response to cold stress and identified three maize genes, *ZmMAPKKK, ZmCLC-D*, and *ZmRLK*, that were possibly involved in the cold response (Yang et al., 2011).

To investigate the regulatory roles of miRNAs (microRNAs) in cold tolerance, Aydinoglu (2020) studied the miRNome (miRNA microarray) in seedlings of the maize hybrid ADA313 that were treated with cold temperature. In this study, 24, 6, and 20 miRNAs were specifically differentially regulated in the meristem, the elongation zone, and the mature zone by cold stress, respectively. This study highlighted the importance of miRNAs in the maize response to cold stress. Combined with large-scale bioinformatic analysis, Zhou et al. (2022) examined the transcriptome changes in seedlings of the inbred lines B73, Mo17, and W22 and the F_1_ hybrids with all three combinations (B73xMo17, W22xB73, and W22xMo17) in response to cold or heat stress. They identified many stress-related DEGs among the different maize genotypes and assigned these expression changes to cis-or trans-regulatory mechanisms using the F_1_ hybrid data. Their study answered the question of how sequence differences in cis-elements of different genotypes impact a gene's responsiveness to stress, and shed light on the prediction of a plant response to stress by implementing a more sophisticated model construction (convolutional neural networks).

Above transcriptomic analyses indentified tens to thousands of DEGs in individual studies. This astonishing variaiton in the numbers of DEGs indicates that a vast majority of the DEGs can not overlap to each other, leaving an almost impossible task to mine the conserved overlaping genes. In deed, when Sowiński et al. (2020) surveyed 13 independent studies addressing the transcriptomic changes in response to cold stress in maize seedlings at the V2-V5 stages, they found <500 DEGs were reported in more than one study and the rest are specific to individual studies. Furthermore, among the 13 independent studies survied, four of these 13 studies used moderate low temperature stress and the other nine used severe cold stress. By comparing the DEGs, it was found that the transcriptomic changes that occur in response to moderate low tempreture and severe cold stress are fundamentally different (Sowiński et al., 2020). Taken together, the variation in gene expression in different studies can be caused by the genotypes, tissue, developmental stage, experimental design and the strength of cold. Although the results of individual studies varied considerably, the “common genes” shared by several independent projects after carefully eliminate the differences can be attractive candidates for further functional studies.

## Local and Long-Distance Transmitance of Cold Signals

All of the above studies extensively investigated the “responses” of maize to cold, but how does maize actively perceive cold and transmit these signals to other parts of the plant? In rice, the OsCOLD1 protein was found to localize to the endoplasmic reticulum and plasma membrane and promote GTPase activity by interacting with a G-protein. In response to cold stress, COLD1 changes membrane fluidity to mediate extracellular Ca^2+^ influx and cytosolic Ca^2+^ concentration, and the altered Ca^2+^ concentration acts as a secondary messenger to regulate downstream genes (Ma et al., 2015b). Thus, COLD1 is thought to be the first cold sensor identified in plants (Shi and Yang, 2015). In maize, ZmSEC14p, a Sec14-like protein, was found to regulate the expression of phosphoinositide-specific phospholipase C in the phosphoinositide signal transduction pathway, which generates the second messengers inositol 1,4,5-trisphosphate and 1,2- diacylglycerol for downstream signal transduction (Wang et al., 2016). Over-expression of *ZmSEC14p* results in the up regulation of some cold-responsive genes such as *CBF3*, suggesting its important role in cold signal transduction.

**Figure 2:**
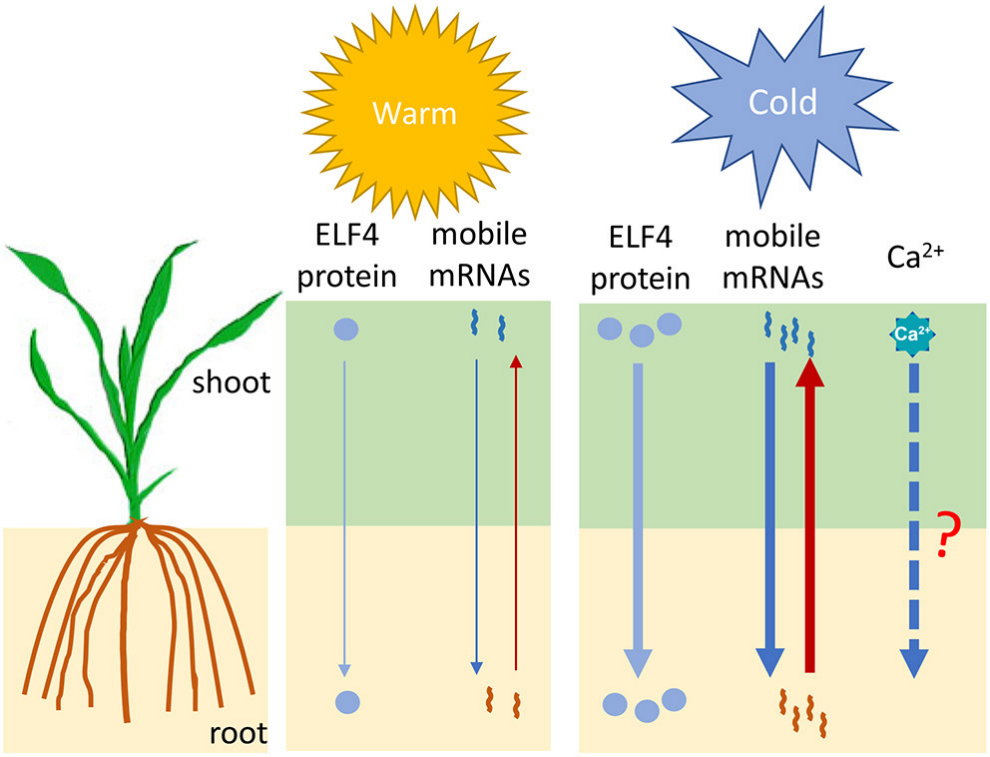
A proposed model for the long-distance regulation of cold signaling by the ELF4 protein and mobile mRNAs. Full and dashed arrows indicate known and potential long-distance signaling molecules, respectively. The thickness of the arrows indicates the relative strength of the signal. ELF4, Early flowering 4.

The above studies, together with the findings in the ICE-CBF-COR pathways, lay important foundations for understanding the cold signal transduction pathway within a local cell; however, the way in which plants transmit cold signals to other parts of the plant is largely unknown. One of the possibilities involves the long-distance signaling system. The phloem sap contains plant hormones, small peptides, transcription factors, and various types of RNA molecules (mRNAs, miRNAs, and tRNAs) that can travel long distances to the other parts of the plant. These molecules, as potential signaling factors, regulate plant growth and development and the stress response (Xia et al., 2018). Wang et al. (2020b) developed a watermelon–bottle gourd heterografting system to identify the mobile mRNAs under both control and cold conditions. Their results showed that cold stress significantly enhances the mobility of mRNAs in the phloem (Figure 2). In particular, some of the scion-delivered mobile mRNAs in the rootstock are derived from some well-known genes related to osmotic adjustment and cold tolerance, while mRNA that moves in the opposite direction includes transcripts from genes related to ABA-activated signaling. In another study, Chen et al. (2020) found that the *Arabidopsis* clock component ELF4 (EARLY FLOWERING 4) protein showed long-distance movement from the shoot to the root through the vascular system to control the root clock in a temperature-dependent manner, and low temperatures favored ELF4 mobility (Figure 2). Hence, ELF4 has been suggested to a mobile long-distance cold signal that establishes a shoot-to-root communication for temperature information. In addition to ELF4 protein and mobile mRNAs, Ca^2+^ has also been implicated as a long-distance signal for water trafficking and defense signaling (Shkolnik et al., 2018; Toyota et al., 2018). A previous study showned that the AtMIZ1 (Mizu-Kussey 1) protein regulates AtECA1 (ER-type CA^2+^-ATPase 1) to generate the long-distance phloem-mobile Ca^2+^ signal for water trafficking (Shkolnik et al., 2018). Interestingly, both *AtMIZ1* and *AtECA1* were found to be responsive to cold treatment (He et al., 2016; Sharma et al., 2018). Despite the involvement of Ca^2+^ in local cold signaling, it is unknown whether Ca^2+^ also functions as a long-distance cold signal through the MIZ1-ECA1- Ca^2+^ pathway (Figure 2). These studies clearly show that long-distance signaling is involved in the response to cold. One very recent study shows that monocotyledonous plants are also capable of being grafted (Reeves et al., 2021), and discoveries related to cold-induced long-distance signaling in maize can be expected soon through use of this powerful method.

## Conclusion

Cold tolerance is an important breeding objective in the pursuit of high productivity and better environmental compliance. Cold tolerance research in maize is of great significance to stabilize yield and enhance food security by broadening the geographical regions in which maize can be cultivated. Cold tolerance in maize is a complex trait and is a cumulative function of many physiological and molecular pathways. Significant achievements have made in studying the physiology of maize cold tolerance, but much remains to be done to solve the problems that result from cold damage to maize plants. The molecular analyses of maize cold tolerance mechanisms are still insufficient, and many of the current studies do not contribute much to maize biology compared to those in *Arabidopsis* and rice. This could be due in part to the lack of discovery of novel genes that may regulate cold tolerance in maize.

There are large differences in cold tolerance among various maize varieties. Cold tolerant varieties usually have a stronger ability to maintain osmotic pressure, the ROS balance, hormonal homeostasis, mineral nutrient absorbance, and photosynthesis. These physiological characteristics have been widely used to evaluate the cold tolerance of maize varieties in QTL and GWAS analyses. Many of the QTL and GWAS analyses were based on various maize genotypes and populations that express large variations in cold tolerance, but only a few candidate genes have been identified. Further fine mapping and map-based cloning of genes in the genomic regions identified *via* QTL and GWAS analysis is required, and more candidate genes will provide a basis for further understanding the molecular and genetic mechanism of cold tolerance in maize. Furthermore, using QTL mapping and GWAS, molecular markers closely linked to cold tolerance genes can also be identified as resources for MAS (marker assisted selection) of cold tolerant varieties. In addition, with the aid of data used for GWAS analysis, future work can also use GS (genomic selection) to predict cold tolerant varieties in large maize populations.

Transcriptome analysis is a powerful tool that has been used in many studies for the identification of cold-responsive genes. Comparisons of the DEGs detected in different studies have shown little overlap with each other. A few studies integrated QTL, GWAS, RNA-seq, and other methods, which significantly reduced the number of candidate genes for selection, partly because joint analyses in the same study can eliminate the differences that arise from the genotypes used or the experimental design. Future research that integrates more methods, such as phenomic, proteomic, metabolomic, and bioinformatic approaches, may greatly improve the accuracy of identifying cold-regulated genes and provide better candidates that can be further used in molecular breeding.

## Author Contributions

CX and HL conceived the work. XZ drafted the manuscript. HL, CX, and IM made comments and revisions. All authors have read and approved the manuscript.

## Funding

This work was supported by the National Natural Science Foundation of China (NSFC) 32101673 and the Sichuan Science and Technology Program 2021YFYZ0017.

## Conflict of Interest

The authors declare that the research was conducted in the absence of any commercial or financial relationships that could be construed as a potential conflict of interest.

## Publisher's Note

All claims expressed in this article are solely those of the authors and do not necessarily represent those of their affiliated organizations, or those of the publisher, the editors and the reviewers. Any product that may be evaluated in this article, or claim that may be made by its manufacturer, is not guaranteed or endorsed by the publisher.
